# Validating the Accuracy of Parkinson's Disease Clinical Diagnosis: A UK Brain Bank Case–Control Study

**DOI:** 10.1002/ana.27190

**Published:** 2025-01-27

**Authors:** Lazzaro di Biase, Pasquale Maria Pecoraro, Vincenzo Di Lazzaro

**Affiliations:** ^1^ Research Unit of Neurology, Neurophysiology and Neurobiology, Department of Medicine and Surgery Università Campus Bio‐Medico di Roma Rome Italy; ^2^ Operative Research Unit of Neurology Fondazione Policlinico Universitario Campus Bio‐Medico Rome Italy; ^3^ Brain Innovations Lab Università Campus Bio‐Medico di Roma Rome Italy

## Abstract

**Objective:**

Despite diagnostic criteria refinements, Parkinson's disease (PD) clinical diagnosis still suffers from a not satisfying accuracy, with the post‐mortem examination as the gold standard for diagnosis. Seminal clinicopathological series highlighted that a relevant number of patients alive‐diagnosed with idiopathic PD have an alternative post‐mortem diagnosis. We evaluated the diagnostic accuracy of PD comparing the in‐vivo clinical diagnosis with the post‐mortem diagnosis performed through the pathological examination in 2 groups.

**Methods:**

In this retrospective case–control study, patients and healthy subjects who consented to the post‐mortem pathological diagnosis at the UK Brain Bank were consecutively enrolled from the UK Brain Bank. Medical records were reviewed to classify participants and performance metrics were further calculated using neuropathological diagnosis as the gold standard.

**Results:**

Four thousand five hundred seventy one subjects were eligible for the study. The clinical diagnosis group was: 1,048 Parkinson's patients and 1,242 healthy subjects. Pathology diagnosis group were: 996 Parkinson's patients and 1,288 subjects with no post‐mortem abnormality. For the group of clinical diagnosis, PD diagnosis showed: sensitivity of 99%, specificity of 86%, accuracy of 90.96%, F1‐Score 0.89, and a receiver operating characteristics area under the curve (ROC AUC) 0.925 (SE ± 0.006) [95% confidence interval [CI]: 0.913, 0.937], 𝑝<0.001. In this group, the most frequent pathology diagnosis among clinically misdiagnosed PD (false positive) patients was dementia with Lewy bodies (19.4%). Conversely, the most frequent clinical diagnosis among PD missed clinical diagnosis (false negative) patients was Alzheimer's disease (18.5%).

**Interpretation:**

Our findings confirm a still significant diagnostic error and emphasize the need for more fine and homogeneous criteria to classify idiopathic Parkinson's patients correctly. ANN NEUROL 2025;97:1110–1121

Despite updates of diagnostic criteria and several studies aiming to better define underlying pathophysiological mechanisms, the accuracy of in‐vivo diagnosis of Parkinson's disease (PD) remains unsatisfying.^1^ To date, clinical criteria for in‐vivo diagnostic certainty are still unmet, requiring pathological confirmation for a conclusive diagnosis.^2^

The gold standard is the post‐mortem examination,^3^ unveiling the concomitant depletion of dopaminergic neurons in the substantia nigra and deposition of Lewy bodies in a predictable fashion, known as Lewy pathology.^4^

The 1988 United Kingdom Parkinson's Disease Society Brain Bank (UKPDSBB) criteria are considered the gold standard for the clinical diagnosis of PD and involve a 3‐step process: (1) Diagnosis of parkinsonism: bradykinesia along with 1 or more among rest tremor, rigidity, or postural instability. (2) Exclusion criteria: rule out other potential causes of parkinsonism. (3) Supportive criteria: 3 or more supportive criteria such as unilateral onset, persistent asymmetry, marked positive response to levodopa (70–100%), response duration to levodopa of 5 years or more, levodopa‐induced chorea, rest tremor, progression of the disease and clinical course of at least 10 years must be present.^1^, ^5^, ^6^

Gelb et al^2^ of 1999 require at least 2 cardinal features among rest tremor, bradykinesia, rigidity or unilateral onset, and at least 1 among tremor and bradykinesia. Atypical features must be excluded, and a sustained levodopa or dopamine agonist response is specifically required.

Gelb criteria distinguish 3 diagnostic certainty levels: (1) Possible PD: 2 cardinal features (1 being tremor or bradykinesia), symptom duration of less than 3 years, and no need for a levodopa response. (2) Probable PD: 3 cardinal features, absence of atypical features for at least 3 years, and a proven levodopa response. (3) Definite PD: confirmed only by neuropathological examination.^2^

Applying these criteria, the possible diagnosis increases sensitivity, while the probable one augments specificity.^1^

Diagnostic criteria for PD proposed in 2015 by the International Parkinson and Movement Disorders Society (MDS) aimed to mimic the diagnostic process of movement disorders experts and augment both reproducibility and accuracy.^7^ MDS criteria enrich the diagnostic process introducing non‐motor symptoms, prodromal PD, and ancillary tests such as olfactory and nuclear medicine assessments.^1^, ^8^, ^9^ These criteria establish 2 levels of diagnostic certainty: (1) Clinically Established PD: absence of exclusion criteria, presence of at least 2 supportive criteria, and no red flags, with high specificity and low sensitivity. (2) Clinically Probable PD: absence of exclusion criteria, red flags counterbalanced by supportive criteria, balancing sensitivity and specificity.

These criteria showed excellent sensitivity and specificity, but movement disorder experts' diagnosis still remains the gold standard for PD diagnosis during life.^10^, ^11^

The present study aims to validate the accuracy of PD clinical diagnosis 2‐fold.

The first one is to evaluate the diagnostic accuracy from alive‐diagnosed PD patients, and dissect pathological diagnoses of clinically‐misdiagnosed patients (false positive to clinical diagnosis), enrolling a group of alive clinically‐diagnosed PD patients and healthy subjects (HS).

The second scope is to investigate the percentage of PD missed diagnosis during life (false negative to clinical diagnosis), by choosing a group of pathology‐proven PD patients and subjects with no post‐mortem pathological finding.

## Methods

The present work follows the Standards for Reporting of Diagnostic Accuracy Studies guidelines.^12^

In this retrospective case–control study, patients and HS who consented to the post‐mortem pathological diagnosis at the UK Brain Bank were consecutively enrolled from January 01, 1972 to December 31, 2019. The provision of data from the UK Brain Bank used in this study was provided with support from the Brains for Dementia Research (BDR) program, jointly funded by Alzheimer's Society UK and Alzheimer's Society. The Campus Bio‐Medico University of Rome's local ethics committee granted its approval for the study.

In the present study, we analyzed post‐mortem data, therefore informed consent was not possible to be obtained for the specific purpose of the present study. However, all the enrolled patients have provided their consent during life to analyze their brain tissue along with their clinical data for each Brain bank of the UK Brain Bank Network. Brain banks from the UK Brain Bank Network that provided data for the present study were Multiple Sclerosis and Parkinson's Tissue Bank, Newcastle Brain Tissue Resource, London Neurodegenerative Disease Brain Bank, Queen Square Brain Bank for Neurological Disorders, Cambridge Brain Bank, Oxford Brain Bank, South West Dementia Brain Bank, Manchester Brain Bank, Edinburgh Brain Bank, and Sheffield Brain Tissue Bank. The data that support the findings of this study are available from the UK Brain Bank. Restrictions apply to the availability of these data, which were used under license for this study.

### Eligibility Criteria

Medical records of patients from the United Kingdom Brain Bank (UKBB) were systematically reviewed by the authors.

Concerning Group 1, subjects with a clinical diagnosis of PD during life, presenting with parkinsonian syndrome, diagnosed in line with the current diagnostic criteria at the moment of the diagnosis, and encoded in the database with the International Classification of Diseases (ICD)‐10 diagnosis code for PD (code G20) were enrolled as PD patients, while subjects identified in the database with the code “No abnormality detected (Present) (+NAD)” and without the diagnosis of PD were labeled as HS.

For Group 2, subjects with a pathological diagnosis of PD and no finding of possible other parkinsonisms from the UK Brain Bank were classified as PD cases, conversely subjects with no detected post‐mortem abnormality and identified in the database with the code “No abnormality detected (Present) (+NAD)” were HS. Each case from both groups was further reviewed by the authors to confirm the assigned diagnostic category.

Neuropathological assessment of all cases followed the standards of UK Brain Bank protocols.

### Statistical Analysis

After data review, subjects from both groups were labeled as true positives (TP) and negatives (TN), and false positives (FP) and negatives (FN). The confusion matrix was evaluated for Groups 1 and 2.

For Group 1, we compared the clinical diagnosis during life to the corresponding post‐mortem pathological finding for each subject. A subanalysis of Group 1 assessed the accuracy of clinical PD diagnosis at both early (clinical diagnosis <3 years from symptom onset) and late (clinical diagnosis <3 years from death) disease timepoints. Conversely, for Group 2, we compared the post‐mortem pathological diagnosis to the corresponding clinical diagnosis in life for each subject. A subanalysis of Group 2 was performed by clustering FN subjects based on the presence or absence of copathology at post‐mortem examination, with a detailed evaluation of their corresponding clinical diagnoses during life to explore potential patterns of clinical misdiagnosis associated with isolated PD pathology or PD with copathologies.

Subsequently, diagnostic performance metrics were established: accuracy of clinical diagnosis, sensitivity, specificity, positive predictive value (PPV), negative predictive value (NPV), diagnostic odds ratio (DOR), positive likelihood ratio (LR+), negative likelihood ratio (LR−), false positive rate (FPR), false negative rate (FNR), false omission rate (FOR), false discovery rate (FDR), and F1‐Score.

All pathological findings and clinical misdiagnoses were further respectively characterized for FP from Group 1 and FN from Group 2.

For Group 1, to further evaluate the discriminative performance of clinical diagnosis, receiver operating characteristic (ROC) curves were plotted following the timeline of PD diagnostic criteria publication for 1972–88, 1989–98, 1999–2014, 2015–19, and for the entire timespan from 1972 to 2019. The ROC curve for the global 1972–2019 period was plotted through average values of sensitivity and specificity. Years of publication of UKBB criteria (1988), Gelb criteria (1999), and MDS criteria (2015) were used as references to estimate the area under the curve (AUC) and assess the accuracy of clinical diagnosis during these timespans. The AUC was computed through the trapezoidal rule for each ROC curve. Interpretation of AUC was based on the following scoring system: 1.0: perfect test, 0.99–0.90: excellent test, 0.89–0.80: good test, 0.79–0.70: fair test, 0.69–0.51: poor test, and 0.50 or lower: fail.^13^ The standard error (SE) and 95% confidence intervals (CI) with *p*‐value were further estimated for each AUC. The significativity level was set at *p* < 0.05.

SPSS (IBM Corp., Armonk, NY) was used as the primary tool for statistical analysis and computation of AUC, SE, 95% CI, and *p*‐values. Python (Python Software Foundation, Wilmington, DE) was the primary software for visualizations and graphical plotting of ROC curves.

## Results

### Study Population and Data Source

Data from 13,343 patients from the UKBB Network were screened. Authors systematically reviewed medical records, reported clinical and pathological diagnoses of all subjects, and found 8,773 cases not fulfilling eligibility criteria. After medical record screening, 4,571 subjects from the UKBB Network were eligible for inclusion and were further grouped according to clinical or pathological diagnosis.

Group 1 is composed of 1,048 patients who received a clinically alive diagnosis of PD and 1,242 HS who did not receive a diagnosis of PD during life or other pathologies.

Out of 1,048 alive‐diagnosed PD subjects, 61.2% were males (n = 642) with an average age at death of 75 (SD ± 17), while the average age at death of enrolled females was 76 (SD ± 16). Out of 1,242 HS, 54.6% (n = 679) were males with an average age at death of 75 (SD ± 17), while the average age at death of enrolled females was 75 (SD ± 17).

**Figure 1:**
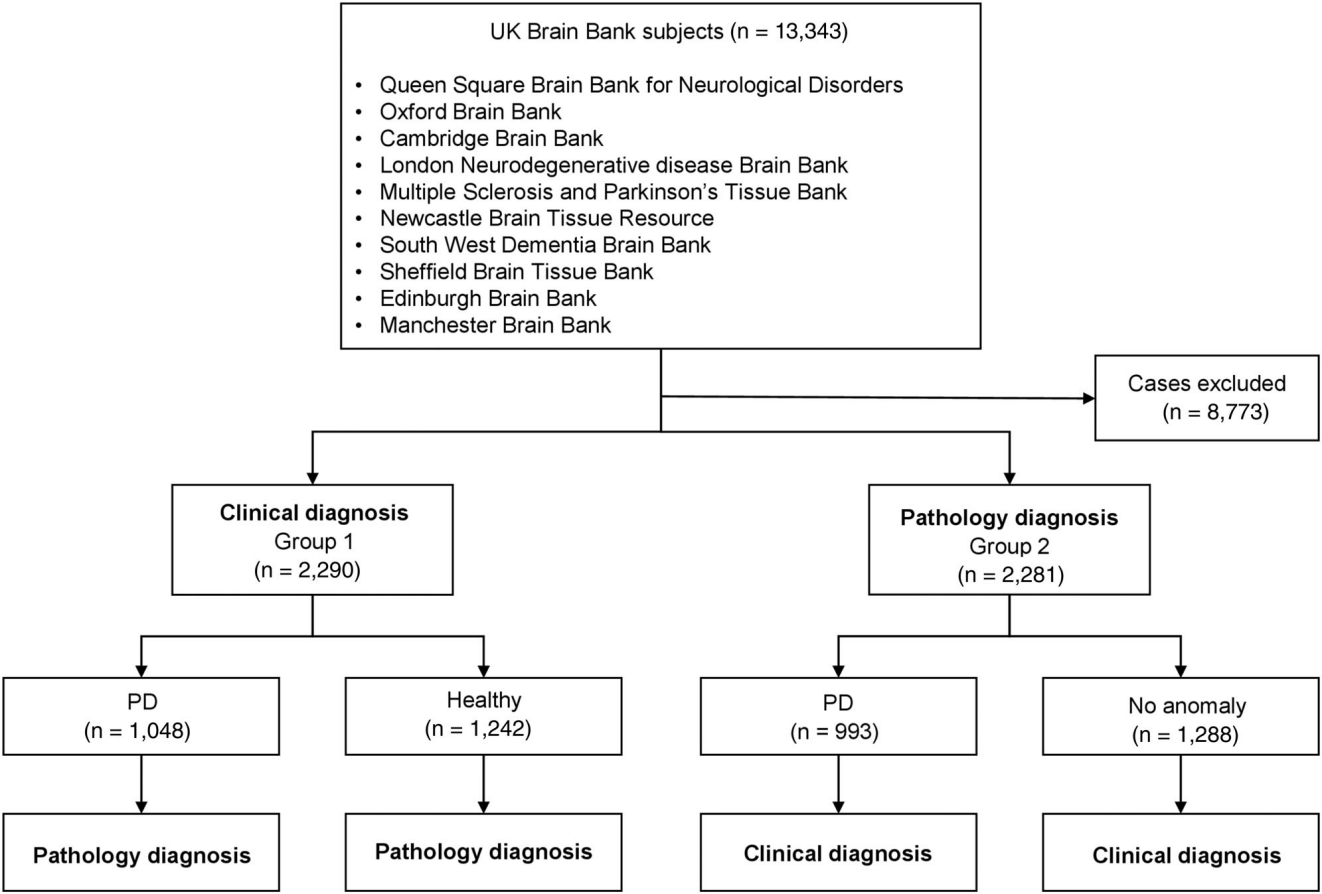
Flowchart of enrollment process of study population and data analysis.

Group 2 is represented by 993 patients who received post‐mortem pathological diagnosis of PD and 1,288 subjects with no post‐mortem pathological anomalies. Figure 1 shows the flowchart of the enrollment process of the study population and data analysis.

Out of 993 pathology‐diagnosed PD subjects, 60.8% were males (n = 604) with an average age at death of 73.7 (SD ± 19), while the average age at death of enrolled females was 73.6 (SD ± 19). Out of 1,288 HS, 58.6% (n = 755) were males with an average age at death of 73.6 (SD ± 19), while the average age at death of enrolled healthy females was 73.6 (SD ± 19).

Tables 1 and 2 summarize data distribution and demographic characteristics of PD and HS from each Brain Bank for respectively Groups 1 and 2.

**Table 1:** Data Source Distribution and Demographic Characteristics of Group 1 from UK Brain Bank Network

Brain Bank	PD			HS		
	No. (%)	Mean Age at Death (yr ± SD)	Gender (M)	No (%)	Mean Age at Death (yr ± SD)	Gender (M)
Queen Square Brain Bank for Neurological Disorders	99 (9.4%)	76 (SD ± 17)	59 (5.6%)	‐		
Oxford Brain Bank	64 (6.1%)	76 (SD ± 17)	43 (4.1%)	236 (19%)	76.3 (SD ± 16.3)	123 (9.9%)
Cambridge Brain Bank	98 (9.3%)	76 (SD ± 16)	49 (4.7%)	215 (17.3%)	76.2 (SD ± 16.4)	117 (9.4%)
London Neurodegenerative disease Brain Bank	105 (10%)	75 (SD ± 17)	58 (5.5%)	315 (25.2%)	76 (±16.8)	161 (13%)
Multiple Sclerosis and Parkinson's Tissue Bank	513 (49%)	76 (SD ± 16)	318 (30.3%)	46 (3.7%)	78.5 (SD ± 14.1)	21 (1.7%)
Newcastle Brain Tissue Resource	112 (10.7%)	76 (SD ± 16.5)	80 (7.6%)	71 (5.7%)	76.1 (SD ± 16.3)	36 (2.9%)
South West Dementia Brain Bank	31 (3%)	76 (SD ± 16)	18 (1.7%)	102 (8.2%)	76.3 (SD ± 16.4)	52 (4.2%)
Sheffield Brain Tissue Bank	1 (0.1%)	77 (SD ± 0)	‐	‐		
Edinburgh Brain Bank	7 (0.6%)	76.5 (SD ± 16)	4 (0.4%)	153 (12.3%)	75 (SD ± 17.1)	125 (10%)
Manchester Brain Bank	18 (1.7%)	76 (SD ± 16)	13 (1.2%)	105 (8.4%)	76.3 (SD ± 16.3)	44 (3.5%)
Total no (%)	1,048 (100%)	76 (SD ± 17)	61.2% (642)	1,242 (100%)	76.3 (SD ± 17)	54.6% (679)

F = female; HS = healthy subjects; M = male; PD = Parkinson's disease.

**Table 2:** Data Source Distribution and Demographic Characteristics of Group 2 from UK Brain Bank Network

Brain Bank	PD			HS		
	No. (%)	Mean Age at Death (yr ± SD)	Gender (M)	No. (%)	Mean Age at Death (yr ± SD)	Gender (M)
Queen Square Brain Bank for Neurological Disorders	131 (13.2%)	73.5 (SD ± 19.2)	85 (8.55%)	10 (0.8%)	73.5 (SD ± 9.5)	1 (0.07%)
Oxford Brain Bank	80 (8%)	73.2 (SD ± 19.4)	46 (4.6%)	350 (27.1%)	73.2 (SD ± 19.5)	193 (15%)
Cambridge Brain Bank	89 (9%)	73.6 (SD ± 19.3)	45 (4.5%)	155 (12%)	73.5 (SD ± 19.3)	82 (6.4%)
London Neurodegenerative disease Brain Bank	67 (6.7%)	73.5 (SD ± 19.3)	35 (3.5%)	289 (22.4%)	73.8 (SD ± 19.3)	165 (12.8%)
Multiple Sclerosis and Parkinson's Tissue Bank	512 (51.5%)	73.7 (SD ± 19)	317 (31.9%)	64 (5%)	73.6 (SD ± 19.2)	30 (2.3%)
Newcastle Brain Tissue Resource	55 (5.5%)	73.2 (SD ± 19.5)	40 (4%)	81 (6.3%)	73.3 (SD ± 19.3)	48 (3.7%)
South West Dementia Brain Bank	34 (3.4%)	73.4 (SD ± 17.5)	22 (22.1%)	85 (6.6%)	77.8 (SD ± 18)	53 (4.1%)
Sheffield Brain Tissue Bank	1 (0.1%)	77 (SD ± 0)	‐	‐		
Edinburgh Brain Bank	9 (0.9%)	71.8 (SD ± 19.3)	5 (0.5%)	228 (17.7%)	72.2 (SD ± 20)	172 (13.3%)
Manchester Brain Bank	15 (1.5%)	70.9 (SD ± 19.9)	9 (0.9%)	26 (2%)	72.1 (SD ± 19.2)	11 (0.8%)
Total no (%)	993 (100%)	73.4.(SD ± 19)	60.8% (604)	1,288 (100%)	73.6 (SD ± 19)	58.6% (755)

F = female; HS = healthy subjects; M = male; PD = Parkinson's disease.

### Group 1: Clinical Diagnosis

Out of 1,048 subjects with a clinically alive diagnosis of PD, 852 cases were confirmed by the anatomopathological investigation postmortem and were labeled as TP, while 196 were not confirmed by the survey postmortem pathology and were considered FP. Subsequently, out of 1,242 alive healthy subjects, 11 were positive for pathological markers of PD and were labeled as FN, while 1,231 had no post‐mortem alteration and were considered TN.

The resulting sensitivity was 99%, specificity 86%, PPV 81%, NPV 99%, accuracy 90.96%, DOR 486.46, LR+ 7.19, LR**−** 0.01, FPR 14%, FNR 1%, FOR 1%, FDR 19%, and the F1‐Score 0.89. Table 3 cross‐tabulates the confusion matrix for this cohort.

**Table 3:** Confusion Matrix for Group 1

	Pathological Diagnosis					
	PD	Not PD				
Clinical Diagnosis						
PD	TP 852	FP 196	Total 1,048	PPV 81%	FDR 19%	Acc 90.96%
HS	FN 11	TN 1,231	Total 1,242	FOR 1%	NPV 99%	
	Total 863	Total 1,427	Total 2,290			
	Sen 99%	FPR 14%	LR+ 7.19	DOR 486.46	F1‐S 0.89	
	FNR 1%	Spec 86%	LR− 0.01			

Acc = accuracy; DOR = diagnostic odds ratio; FN = false negative; FNR = false negative rate; FP = false positive; FPR = false positive rate; HS = healthty subjects; LR**−** = negative likelihood ratio; LR+ = positive likelihood ratio; NPV = negative predictive value; PD = Parkinson's disease; PPV = positive predictive value; TN = true negative; TP = true positive.

Among 1,048 clinically diagnosed PD patients, 7.8% (n = 81) were diagnosed within 3 years of symptom onset, and 5.9% (n = 62) within 3 years before death.

For the early‐diagnosis subgroup, sensitivity reached 80.7%, specificity 97.24%, PPV 56.8%, NPV 99.11%, FPR 2.76%, FNR 19.3%, FDR 56%, LR+ 29.19, LR**−** 0.2, FOR 1%, DOR 147.08, F1‐Score 0.67, and accuracy 96.52%. In contrast, the late‐diagnosis subgroup showed a sensitivity of 78.4%, specificity of 98.2%, PPV of 64.5%, NPV of 99.11%, FPR 1.76%, FNR 21.57%, FDR 35.4%, LR+ 44.67, LR**−** 0.22, FOR 1%, DOR 203.47, F1‐Score 0.71, and accuracy 97.47%. Tables S1 and S2 summarize the confusion matrices for both subgroups.

Out of 196 FP subjects, 53.6% received a single pathological diagnosis, 37.2% a combination of 2 diagnoses, and 9.2% a combination of 3 diagnoses. Dissecting FP group, 19.4% had an isolated diagnosis of dementia with Lewy bodies (DLB), 9.2% multiple system atrophy (MSA), 7.6% Alzheimer's disease (AD), 6.6% vascular encephalopathy (VE), 4.6% another isolated diagnosis, 2% frontotemporal dementia (FTD), 1.5% progressive supranuclear palsy (PSP), 1.5% corticobasal degeneration, 1% motor neuron disease (MND).

Differently, when copathology was found, the most common mixed dual diagnoses were AD + VE and MND + PSP.

**Figure 2:**
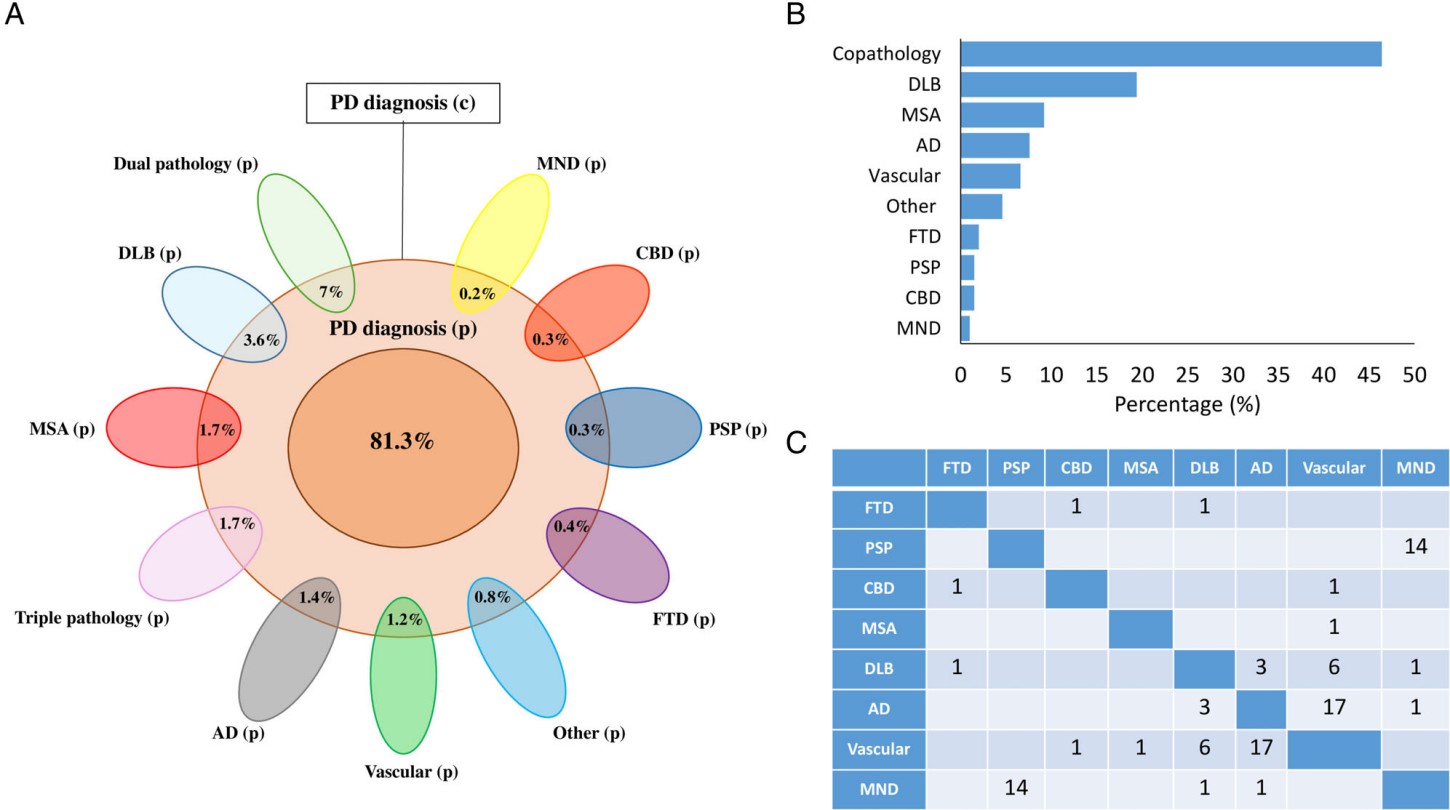
Pathology diagnoses of Group 1. (A) Relative representation of pathological diagnoses from all participants of Group 1. (c) Stands for clinical diagnosis. (p) Stands for pathological diagnosis. (B). Percentage of post‐mortem pathological diagnoses for only false positive (FP) cases. (C) FP subjects with a combination of 2 pathological diagnoses. AD = Alzheimer's disease; CBD = corticobasal degeneration; DLB = dementia with Lewy bodies; FTD = frontotemporal dementia; MND = motor neuron disease; MSA = multiple system atrophy; PSP = progressive supranuclear palsy. [Color figure can be viewed at www.annalsofneurology.org]

Figure 2 summarizes percentages of pathological diagnoses (A and B) and FP subjects with a combination of 2 pathological diagnoses (C).

### Group 2: Pathological Diagnosis

Out of 993 subjects with post‐mortem pathological diagnosis of PD, 842 had received a clinically alive diagnosis of PD and were labeled as TP, while 151 had not received a clinical diagnosis of PD during life and were considered FN. Among 1,288 subjects with no post‐mortem alteration, 5 had received a clinical diagnosis of PD during their lifetime and were labeled as FP, while 1,283 that were considered healthy subjects had no sign of PD at the pathology examination and were considered as TN.

The resulting sensitivity was 84.79%, specificity 99.61%, PPV 99.41%, NPV 89.47%, accuracy 93.16%, DOR 1430.84, LR+ 218.43, LR**−** 0.15, FPR 0.39%, FNR 15.21%, FOR 10.53%, FDR 0.59%, and the F1‐Score was 0.92. Table 4 cross‐tabulates the confusion matrix for this cohort.

**Table 4:** Confusion Matrix for Group 2

	Pathological Diagnosis					
	PD	Not PD				
Clinical Diagnosis						
PD	TP 842	FP 5	Total 847	PPV 99.41%	FDR 0.59%	Acc 93.16%
HS	FN 151	TN 1,283	Total 1,434	FOR 10.53%	NPV 89.47%	
	Total 993	Total 1,288	Total 2,281			
	Sen 84.79%	FPR 0.39%	LR+ 218.43	DOR 1,430.84	F1‐S 0.91	
	FNR 15.21%	Spec 99.61%	LR− 0.15			

Acc = accuracy; DOR = diagnostic odds ratio; FN = false negative; FNR = false negative rate; FP = false positive; FPR = false positive rate; HS = healthty subjects; LR**−** = negative likelihood ratio; LR+ = positive likelihood ratio; NPV = negative predictive value; PD = Parkinson's disease; PPV = positive predictive value; TN = true negative; TP = true positive.

Out of 151 FN subjects, 88% received a single clinical diagnosis, while 9.2% and 2.6% respectively had 2 and 3 diagnoses.

**Figure 3:**
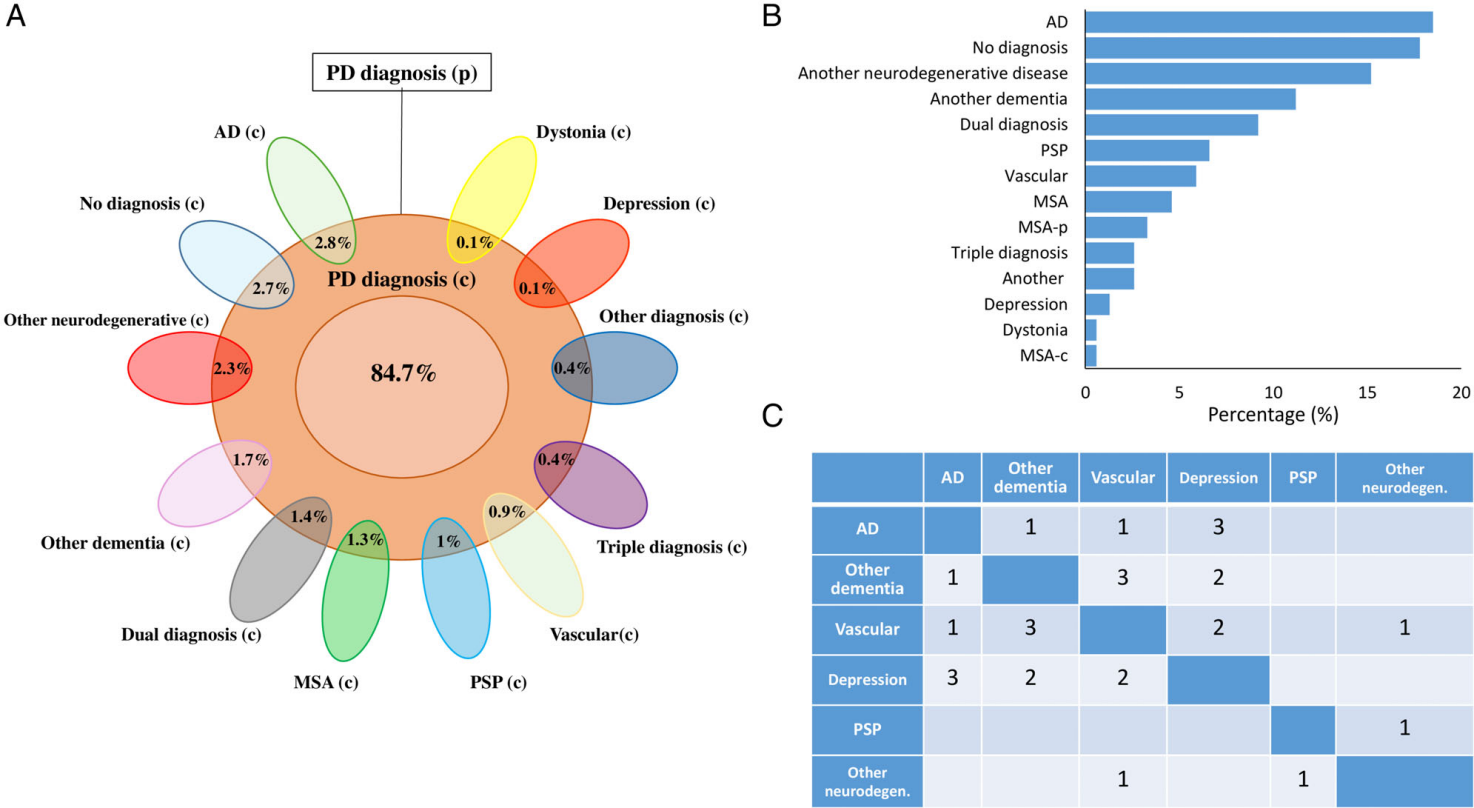
Clinical diagnoses of Group 2. (A) Relative representation of clinical diagnoses from all participants of Group 2. (p) Stands for pathological diagnosis. (c) Stands for clinical diagnosis. (B) Percentage of clinical misdiagnoses for only FN. (C) FN subjects with a combination of 2 in‐vivo clinical diagnoses. AD = Alzheimer's disease; CBD = corticobasal degeneration; DLB = dementia with Lewy bodies; FTD = frontotemporal dementia; MND = motor neuron disease; MSA = multiple system atrophy; MSA‐C = multiple system atrophy‐cerebellar type; MSA‐P = multiple system atrophy‐parkinsonian type; PSP = progressive supranuclear palsy. [Color figure can be viewed at www.annalsofneurology.org]

Among 151 clinically misdiagnosed subjects, AD had been diagnosed in 18.5% of cases, no diagnosis in 17.8%, another isolated neuro‐degenerative disease in 15.2%, other isolated dementia in 11.2%, PSP in 6.6%, vascular disease in 5.9%, MSA in 4.6%, MSA‐P (parkinsonian type) in 3.3%, another isolated diagnosis in 2.6%, depression in 1.3%, dystonia in 0.6%, and MSA‐C (cerebellar type) in 0.6% of cases. Among FN subjects with dual clinical diagnoses, the most common in‐vivo dual clinical diagnoses were depression with AD and other dementia with VE. Figure 3 summarizes percentages of clinical diagnoses (A and B) and FN subjects with a combination of 2 clinical diagnoses (C).

#### Subgroup 1: Isolated Pathological Diagnosis of PD

The post‐mortem examination diagnosed an isolated PD (presence of diffuse Lewy body disease without any other pathological findings at autopsy) in 37.1% (n = 56) of all FN cases. A total of 34.4% of FN cases with an isolated PD at autopsy had only of clinical diagnosis in life, while 2.6% had 2 clinical diagnoses. The isolated clinical diagnoses made during life were other specified degenerative disease of the nervous system (28.6%), no abnormality detected (25%), PSP (12.5%), MSA (10.7%), AD (3.6%), other dementia (3.6%), VE (1.8%), depression (1.8%), dystonia (1.8%), MSA‐P (1.8%), and MSA‐C (1.8%), and fragile X syndrome (1.8%). For the dual clinical diagnosis group, the combinations were AD and other dementia (1.8%); other dementia and depression (1.8%), and AD and depression (1.8%). For the triple diagnoses group, the only combination was AD with depression and focal brain injury.

#### Subgroup 2: Pathological Diagnosis of PD with Copathology

Among FN cases 62.9% had at least 1 pathological diagnosis at the post‐mortem examination alongside PD.

Isolated clinical diagnosis during life was found in 53.6%: AD (14.0%), other dementia (8.4%), no diagnosis (7.9%), other specified degenerative disease of the nervous system (5.5%), VE (4.5%), and other diagnoses (3.9%), MSA‐P (2.2%), PSP (1.7%), depression (0.7%), MSA (0.7%). Two clinical diagnoses were present in 6.6% of FN cases, including the following combinations: AD and depression (1.3%), other dementia and VE (1.3%), AD and VE (0.7%), AD and other dementia (0.7%), VE and depression (0.7%), other dementia and depression (0.7%), PSP and other specified degenerative disease of the nervous system (0.7%), and VE and other specified degenerative disease of the nervous system (0.7%). Three clinical diagnoses were identified in 2.6% of FN cases, and were: AD, other dementia, and VE (0.7%); AD, other dementia, and another specified degenerative disease of the nervous system (0.7%); AD, other dementia, and depression (0.7%); and other dementia, VE, and depression (0.7%).

From the pathological detail of 151 FN brains, 37.1% had 1 additional pathological diagnosis, 21.2% had 2, 4.0% had 3, and 0.7% had 4 coexisting pathological findings alongside post‐mortem PD. From a pathological standpoint, the most frequent additional diagnoses to diffuse Lewy body disease were:

One post‐mortem diagnosis: AD neuropthalogic changes (ADNPC) (72%), with the most common clinical diagnoses during life being AD (45%), followed by “no abnormaility detected” (code [+NAD]) (25%), followed by vascular or unspecified dementia (12.5%), PSP (2.5%), and other combinations in the remaining cases.

Two post‐mortem diagnoses: ADNPC and VE (43.7%), where the most frequent clinical diagnoses were unspecified dementia (28.6%), AD (21.4%), and vascular dementia (21.4%), and other combinations in the remaining cases.

Three post‐mortem diagnoses: ADNPC, VE, and cerebral amyloid angiopathy (CAA) (83.3%), where clinical diagnoses during life included unspecified cerebrovascular disease (40%), AD with vascular dementia (20%), AD combined with stroke (20%), and other combinations in the remaining cases.

Four post‐mortem diagnoses: ADNPC, VE, CAA, and non‐active demyelination (100%), with clinical diagnosis during life being unspecified dementia (100%).

Combinations of copathologies identified in post‐mortem diagnosis of PD and corresponding clinical diagnoses during life in FN cases from Group 2 are listed in Table S3.

### Diagnostic Performance Analysis

**Figure 4:**
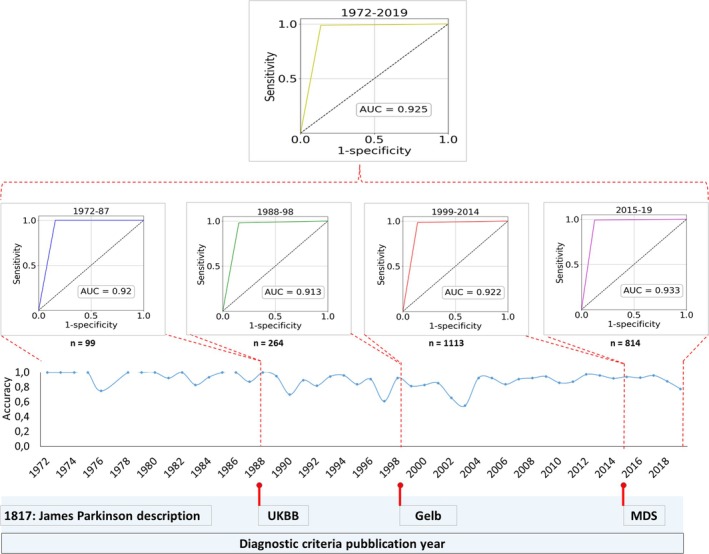
Diagnostic performance analysis for PD clinical diagnosis over time. ROC curves with relative AUC for years between publication of main diagnostic criteria and for the entire timespan from 1972 to 2019 (*upper part*). Trend of accuracy for PD clinical diagnosis from 1972 to 2019 (*lower part*). AUC = area under the ROC curve; MDS = Movement Disorders Society; n = number of patients included; PD = Parkinson's disease; ROC = receiver operating characteristic; UKBB = United Kingdom Brain Bank. [Color figure can be viewed at www.annalsofneurology.org]

Taking diagnostic performances from 1972 to 2019 into account, we first analyzed the trend of accuracy for PD clinical diagnosis over time, as shown in Figure 4.

Worst accuracy performances were found in 1990, 1997, 2002, and 2003 with values of, respectively, 70.3%, 61.1%, 65%, and 55.1%. An accuracy of at least 75% for all the remaining years was found.

Based on the values of sensitivity and specificity of Group 1 from 1972 to 2019, we evaluated the discriminative performance of clinical diagnosis for 1972–87, 1988–98, 1999–2014, 2015–19, and for the entire timespan from 1972 to 2019. We found that from 1972 to 1987 when PD diagnosis was still anchored to James Parkinson's description, the accuracy was 97.92%. Between 1988 and 1998, after the UKBB criteria publication, the accuracy was 87.12%. From 1999 to 2014, after the Gelb criteria publication, we found an accuracy of 90.66%. For 2015–19, after the MDS criteria publication, the accuracy was 92.38%.

ROC AUC (SE) and 95% CI with *p*‐value were computed for the various years across the publication of the diagnostic criteria and for the whole timespan to assess the diagnostic performance over time. ROC curves were further plotted (Fig 4).

For the 1972–87 period, the ROC analysis indicated an AUC of 0.920 (SE ± 0.033) [95% CI: 0.855, 0.986], 𝑝<0.001. For the 1988–98 period, ROC analysis indicated an AUC of 0.913 (SE ± 0.02) [95% CI: 0.875, 0.951], 𝑝<0.001. For the 1999–2014 period, the ROC analysis indicated an AUC of 0.922 (SE ± 0.009) [95% CI: 0.905, 0.939], 𝑝<0.001. For the 2015–19 period, the ROC analysis indicated an AUC of 0.933 (SE ± 0.01) [95% CI: 0.915, 0.952], 𝑝<0.001. Finally, for the full period from 1972 to 2019, the ROC analysis indicated an AUC of 0.925 (SE ± 0.006) [95% CI: 0.913, 0.937], 𝑝<0.001.

## Discussion

In the present case–control pathology‐validated study, we retrospectively compared the accuracy of PD diagnosis through post‐mortem examination over 5 decades.

For the whole period and considering pathology validation as the gold standard, the diagnostic accuracy through in‐vivo clinical examination applying diagnostic criteria of current clinical practice was 90.96% with a sensitivity of 99% and a specificity of 86%.

We found that AUC for clinical diagnosis of PD was “excellent” for all years across the publication of different diagnostic criteria from 1972 to 2019. Overall performance of clinical diagnosis was also definitely “excellent”. Accuracy was globally over 90% before the publication of the first structured diagnostic criteria for PD (UKBB) and after both Gelb and MDS criteria approvals.

The findings of this study underscore the persistent challenges in achieving an accurate in‐vivo diagnosis of PD using clinical criteria alone. Indeed, clinicopathological series evaluating any clinical diagnostic accuracy improvement have been poorly explored and seminal works validating UKPDSBB criteria highlighted that around one‐fourth of patients alive‐diagnosed as PD have an alternative post‐mortem diagnosis,^1^, ^3^, ^6^, ^14^, ^15^ proving the best accuracy with the refined diagnosis of a movement disorders' expert over follow‐up. Additionally, only 1 study directly compared the accuracy of UKPDSBB and Gelb criteria with the neuropathologic assessment as the diagnostic gold standard and highlighted that the UKPDSBB criteria had slightly higher diagnostic accuracy and sensitivity.^16^ A review of medical records of 267 alive‐diagnosed parkinsonism patients from the Queen Square Brain Bank between 2009 and 2019 was performed by Virameteekul et al.^17^ Authors retrospectively applied MDS criteria and found an improvement in clinical diagnostic accuracy, more marked at early disease stages.

By the subanalysis of Group 1, our findings indicate consistently high sensitivity in both early and late PD diagnoses, reflecting the robustness of clinical identification across disease stages. However, specificity, and accuracy were slightly higher in late‐stage diagnoses, suggesting enhanced discriminative performance when diagnosis was in late stage. Specifically, the DOR was higher in the late‐diagnosis subgroup compared to the early one (203.47 vs 147.08): the balance between sensitivity and specificity is more favorable in late‐stage diagnosis, enhancing the likelihood that a positive diagnosis truly reflects the presence of PD.

Nevertheless, the high NPV across both subgroups underscores the strong capacity of the clinical diagnostic criteria to rule out PD, demonstrating their clinical utility throughout different PD diagnostic phases.

In summary, published clinicopathologic series on post‐mortem validation of PD clinical diagnostic criteria documented a broad accuracy range from 69.5% to 96.7%. Diagnosis made by experts and refinement at final disease stages improved diagnostic performance and outperformed current criteria.

In line with Virameteekul et al^17^ who found an AUC range from 0.908 to 0.967 for expert clinical diagnosis, when retrospectively applying MDS criteria, we found an AUC of 0.933 for the timespan after the publication of MDS criteria.

On the diagnostic accuracy level, our findings align broadly with prior research on the topic and confirm a still significant diagnostic error, emphasizing the need for more fine and homogeneous criteria to correctly classify idiopathic PD patients and distinguish them from possible mimics.

The elevated number of false positives (196 for the alive PD diagnosis group) and false negatives (151 for the post‐mortem PD diagnosis group) demonstrates that a more accurate differential diagnosis is mandatory, in particular versus atypical parkinsonisms and dementias. The high sensitivity but relatively lower specificity observed for the clinical PD diagnosis suggests that clinicians may err on the side of over‐diagnosis when faced with parkinsonian symptoms.

The most common isolated neuropathological findings for false positives from our large cohort of alive clinically diagnosed PD subjects were DLB (19.4%), MSA (9.2%), and AD (7.6%). However, copathology was common among false positives with dual pathology represented in 37.2% and triple pathology in 9.2% of cases.

The most common clinical isolated misdiagnosis during life for post‐mortem diagnosed PD patients, ie, false negatives (Group 2), was AD (18.5%), followed by other dementias (11.2%), PSP (6.6%), VE (5.9%), and MSA (4.6%, split in MSA‐P 3.3%, MSA‐C 0.6%, and no better specified MSA 0.7%).

Also in this group, the combination of more diagnoses during life was quite common with dual clinical diagnosis in 9.2% and triple clinical diagnosis in 2.6% of cases. These findings point toward AD and other dementias as a confounding alive diagnosis, in which cognitive problem focus the attention more than the movement ones, and in addition confirm the well‐known difficulty for correct clinical‐based discrimination between PD dementia and DLB.

As confirmed by our results, a comprehensive model for the diagnosis of PD should take also copathology into account: the presence of multiple pathologies is now recognized as the rule rather than the exception in the neurodegeneration field.^18^ Based on our cohort of alive‐diagnosed PD subjects, 73 out of the 196 misdiagnosed patients had a combination of 2 pathological diagnoses and 18 a combination of even 3. Dissecting copathology, we found that the most represented combinations of dual mixed pathologies were VE + AD and MND + PSP. Accordingly, data from 3 community‐based longitudinal cohorts of 1,430 older adults showed that the average PD patient has a median of 3 pathologies and in some cases up to 9 and that the weighted pathology score does not account for the rate of progression when comparing those with versus without clinical PD.^19^ In another retrospective study of 1,647 autopsied individuals, up to 7 different pathologies occurred in 161 combinations, matching pathology and diagnosis of only between 19% and 45%.^20^

In this regard, when approaching to the post‐mortem results in FN subjects, our findings reveal a significant diagnostic challenge in pathology‐proven PD cases with isolated or coexisting pathologies. In particular, those with isolated DLBD (37.1%) were frequently misdiagnosed during life with conditions such as other specified degenerative diseases of the nervous system (28.6%) or labelled as having no abnormality (25%), PSP (12.5%), or MSA (10.7%). Interestingly, co‐pathologies, such as AD or VE were present in the majority of FN cases with multiple post‐mortem findings, which often corresponded to clinical diagnoses of unspecified dementia or vascular dementia. In summary, the results underscore the complexity of clinical diagnosis in PD, particularly in the presence of co‐pathologies, suggesting that the coexistence of vascular or neurodegenerative co‐pathologies alongside diffuse Lewy bodies may skew clinical impressions toward non‐PD syndromes such as parkinsonisms or dementias.

Noteworthy, the latest attempt at PD diagnosis is based on a biological definition with 2 similar but diverging frameworks: (1) the neuronal α‐synuclein disease (NSD) integrated staging system (NSD‐ISS)^21^ and (2) the SynNeurGe model.^22^

According to these pipelines, the biological definition of PD arises from combining: (a) neurodegeneration: evidence of loss of dopaminergic neurons with neuroimaging and (b) pathological α‐synuclein detection: through seed amplification assay (SAA) and (c) genetic risk.

Posing the diagnosis and the definition of the disorder on its underlying biology rather than the description of the syndrome appears as a major advance in the conceptualization of neuronal α‐synuclein disease. The absence of clinical symptoms as disease‐defining pieces for an early diagnosis, as proposed by new biological definition frameworks, seem controversial since both the proportion and timing of phenoconversion are currently uncertain, and diagnosing a “disease” in individuals who will never lifelong experience disease‐related symptoms could lead to ethical concerns.^23^, ^24^ However, up to 151 subjects without in‐vivo parkinsonian symptoms had post‐mortem findings of PD. These subjects could potentially belong to stages 0–1B of the SynNeurGe model or their missed PD diagnosis may have risen from a non‐meticulous neurological examination. This result highlights the urgent need for new biomarkers to screen asymptomatic subjects for early PD detection, in order to follow these people up to the emergence of PD symptoms.

As our understanding of the biological underpinnings of parkinsonian syndromes continues to advance, it will be crucial to integrate these insights into the refinement of clinical diagnostic criteria. Incorporating biomarkers and ancillary tests that can reliably differentiate the various pathological subtypes may enhance the clinician's ability to make an accurate ante‐mortem diagnosis. Ongoing longitudinal studies tracking the progression of clinical features alongside parallel changes in molecular and neuroimaging markers will be essential to further validate and refine the biological definition of PD.

Starting from late 2016, kinetic analysis of SAA fluorescence profiles has enabled the identification of synucleinopathy‐dependent α‐syn fibril conformations, holding potential to differentiate synucleinopathies^25^, ^26^ and refine PD diagnosis with a sensitivity and specificity between 89% and 100%.^27^ In this regard, our data indicate that, while alpha‐synuclein SAAs are promising in the diagnostic toolbox, their relatively recent introduction into research and clinical practice has resulted in a moderate impact on diagnostic accuracy within our cohort, encompassing only the late 2016–19 timespan. Our findings primarily reflect the robustness of traditional clinical criteria, which emphasize a longitudinal approach to diagnosis, maintaining consistent reliability throughout the study period. SAAs have not fundamentally changed the diagnostic process within our cohort, as clinical criteria rely on detailed, long‐term observation of motor and non‐motor symptoms. Consequently, improvements in diagnostic accuracy in our study are more attributable to the refinement of clinical criteria and increasing clinical expertise than to biomarkers usage.

Possible limitations of our Brain Bank‐based study include a bias toward more severe or atypical presentation in the Brain Bank, which should be taken into account for the interpretation and generalization of the results.^10^ Despite the risk of higher clinical misdiagnosis rates, we minimized this bias by including 2 control groups. The assessment by different clinicians without a clear methodological homogeneity for diagnostic criteria and the retrospective nature of the study could have influenced data reporting methods. Nonetheless, the relatively small number of cases diagnosed within 3 years of symptom onset or death may affect generalizability of the results.

While SAAs hold promise for enhancing early PD detection, unravelling potential for a diagnosis at a preclinical stage beyond the capacity of traditional criteria, the clinical criteria's primary strength remains in diagnosing symptomatic rather than preclinical or prodromal cases.^28^ Promoting α‐syn SAAs for a molecular‐based routine workup of synucleinopathies needs to be fully validated, by mandatorily addressing issues related to protocol and recombinant substrate standardization as well as those related to the quantitative response of the technique.

In summary, pathological validation is pivotal to filling the gap of the diagnostic error in PD, as the first step toward new criteria that reflect a comprehensive vision for a biological definition of the disease.

## Conclusion

Despite refinements in criteria and guidelines, clinical diagnosis of PD still suffers from a not satisfying accuracy and specificity, with the post‐mortem validation as the diagnostic gold standard. Identifying more solid in‐vivo biomarkers, improving the characterization of prodromal symptoms and risk factors, and increasing knowledge of etiopathological mechanisms are required steps to augment the diagnostic process. Pathological examination is pivotal for new diagnostic criteria that reflect a comprehensive vision for a biological definition of the disease.

## Author Contributions

L.dB. and V.D.L. contributed to the conception and design of the manuscript. L.dB. and V.D.L. contributed to the acquisition and analysis of the data. L.dB., P.M.P., and V.D.L. contributed to drafting a significant portion of the manuscript and figures.

## Potential Conflicts of Interest

Nothing to report.

## Supporting information


**Table S1.** Confusion matrix for early‐PD subanalysis of Group 1.

**Table S2.** Confusion matrix for late‐PD subanalysis of Group 1.

**Table S3.** Copathologies identified in post‐mortem diagnosis of PD and corresponding clinical diagnoses during life in FN cases from Group 2.

## Data Availability

Individual participant data that underlie the results reported in this article, after de‐identification (text, tables, figures, and appendices), are available from the corresponding author upon reasonable request and after the permission of the UK Brain Bank.
