# Supplementary material for: Validating the Accuracy of Parkinson's Disease Clinical Diagnosis: A UK Brain Bank Case–Control Study
Source: Ann Neurol. 2025 Jan 27;97(6):1110–21. doi: 10.1002/ana.27190 (PMC12082010; doi:10.1002/ana.27190)
Supplement: Supplementary file 1 — Table S1. Confusion matrix for early‐PD subanalysis of Group 1. [file ANA-97-1110-s002.docx]

**Table S1. Confusion matrix for early-PD subanalysis of Group** **1.**

|  | | Pathological diagnosis | |  | | | | |
| --- | --- | --- | --- | --- | --- | --- | --- | --- |
|  |  | PD | Not PD |  |  |  |  |  |
| Clinical diagnosis | Early PD | TP  46 | FP  35 | Total  81 | PPV  56.8% | FDR  56% | | Acc  96.52% |
|  | HS | FN  11 | TN  1231 | Total  1242 | FOR  1% | NPV  99.1% | |  |
|  | | Total  55 | Total  1266 | Total  1323 |  | | |  |
|  |  | Sen  80.7% | FPR  2.76% | LR+  29.19 | DOR  147.08 | | F1-S  0.67 |  |
|  |  | FNR  19.3% | Spec  97.2% | LR-  0.2 |  | | |  |

Abbreviations: PD= Parkinson’s disease; HS= Healthty Subjects; TP= True Positive; FP= False Positive; FN= False Negative; TN= True Negative; FNR= false negative rate; FPR= false positive rate; FPR= False Positive Rate; Positive Predictive Value= PPV; Negative Predictive Value= NPV; FNR= False Negative Rate; LR+= positive likelihood ratio; LR-= negative likelihood ratio; DOR= Diagnostic Odds Ratio.
