# Supplementary material for: Validating the Accuracy of Parkinson's Disease Clinical Diagnosis: A UK Brain Bank Case–Control Study
Source: Ann Neurol. 2025 Jan 27;97(6):1110–21. doi: 10.1002/ana.27190 (PMC12082010; doi:10.1002/ana.27190)
Supplement: Supplementary file 3 — Table S3. Copathologies identified in post‐mortem diagnosis of PD and corresponding clinical diagnoses during life in FN cases from Group 2. [file ANA-97-1110-s003.docx]

Table S3. Copathologies identified in post-mortem diagnosis of PD and corresponding clinical diagnoses during life in FN cases from Group 2.

| **Additional pathological diagnoses to post-mortem PD (n)** | **Percentage relative to FN group (%)** | **Number of cases (n)** | **Pathological diagnoses (%)** | **Clinical diagnoses during life (%)** |
| --- | --- | --- | --- | --- |
| Zero | 37 | 56 | PD (Isolated DLBD) (100%) | Other degenerative diseases of the nervous system (28.6%) |
|  |  |  |  | No abnormality (25%) |
|  |  |  |  | PSP (12.5%) |
|  |  |  |  | MSA (10.7%) |
|  |  |  |  | Unspecified dementia (3.6%) |
|  |  |  |  | AD (3.6%%) |
|  |  |  |  | MSA-p (1.8%) |
|  |  |  |  | MSA-c (1.8%) |
|  |  |  |  | AD, other dementia (1.8%) |
|  |  |  |  | AD, depression, focal brain injury (1.8%) |
|  |  |  |  | Other dementia, depression (1.8%) |
|  |  |  |  | Depression (1.8%) |
|  |  |  |  | Dystonia (1.8%) |
|  |  |  |  | Vascular dementia (1.8%) |
|  |  |  |  | Fragile X syndrome (1.8%) |
| One | 37 | 56 | AD (72%) | AD (45%) |
|  |  |  |  | No abnormality (25%) |
|  |  |  |  | Unspecified/vascular dementia (12.5%) |
|  |  |  |  | AD, TIA/depression/other degenerative diseases of nervous system (5%) |
|  |  |  |  | Other degenerative diseases of the nervous system (7.5%) |
|  |  |  |  | PSP (2.5%) |
|  |  |  |  | AD, other degenerative diseases of nervous system, vascular dementia (2.5%) |
|  |  |  | VE (17.8%) | Vascular dementia (30%) |
|  |  |  |  | No abnormality (30%) |
|  |  |  |  | Cerebrovascular disease, Unspecified dementia (10%) |
|  |  |  |  | MSA-p (10%) |
|  |  |  |  | Unspecified stroke (10%) |
|  |  |  |  | Cerebrovascular disease, depression, anxiety (10%) |
|  |  |  | Unspecified tauopathy (3.6%) | Vascular dementia (50%) |
|  |  |  |  | PSP (50%) |
|  |  |  | CBD (1.8%) | Unspecified dementia (100%) |
|  |  |  | MND (1.8%) | Motoneuron disease (100%) |
|  |  |  | Agyrophilic grain disease (1.8%) | MSA-p (100%) |
|  |  |  | PSP with FTLD (1.8%) | PSP (100%) |
| Two | 21.2 | 32 | AD and VE (43.7%) | Unspecified dementia (28.6%) |
|  |  |  |  | AD (21.4%) |
|  |  |  |  | Vascular dementia/stroke (21.4%) |
|  |  |  |  | Other degenerative diseases of the nervous system (21.4%) |
|  |  |  |  | MSA-p (7.1%) |
|  |  |  | AD and CAA (19%) | Unspecified cerebrovascular disease/dementia (50%) |
|  |  |  |  | AD, stroke (16.6%) |
|  |  |  |  | AD, depression (16.6%) |
|  |  |  |  | Depression (16.6%) |
|  |  |  | AD and chronic arachnoiditis (3.1%) | Other degenerative diseases of the nervous system (100%) |
|  |  |  | AD and meningioma (3.1%) | Unspecified dementia (100%) |
|  |  |  | AD and pontine myelinolysis (3.1%) | Other degenerative diseases of the nervous system (100%) |
|  |  |  | Aging and VE (3.1%) | No abnormality (100%) |
|  |  |  | CBD and VE (3.1%) | PSP (100%) |
|  |  |  | Glioblastoma and unspecified tauopathy (3.1%) | Malignant tumour of the nervous system (100%) |
|  |  |  | Gliomatosis cerebri and hippocampal sclerosis (3.1%) | MSA (100%) |
|  |  |  | Polyglucosan body disease and VE (3.1%) | AD (100%) |
|  |  |  | VE and pontine hemorrhage (3.1%) | No abnormality (100%) |
|  |  |  | Argyrophilic grain disease and CAA (3.1%) | No abnormality (100%) |
|  |  |  | Unspecified tauopathy and VE (3.1%) | Vascular parkinsonism (100%) |
|  |  |  | Chronic traumatic encephalopathy and VE (3.1%) | MSA-p (100%) |
| Three | 4 | 6 | AD, VE, and CAA (83.3%) | Unspecified cerebrovascular disease (40%) |
|  |  |  |  | AD, vascular dementia (20%) |
|  |  |  |  | AD, stroke (20%) |
|  |  |  |  | Unspecified stroke (20%) |
|  |  |  | AD, TDP-43 proteinopathy, and VE (16.6%) | Other degenerative diseases of the nervous system (100%) |
| Four | 0.7 | 1 | AD, VE, CAA, and non-active demyelination (100%) | Unspecified dementia (100%) |

Abbreviations: AD= Alzheimer disease; CAA = cerebral amyloid angiopathy; CBD = corticobasal degeneration; DLBD= diffuse Lewy Body disease; FN= false negative; MND= motoneuron disease; MSA= multiple system atrophy; PSP= progressive supranuclear palsy; TIA= transient ischemic attack; VE = vascular encephalopathy.
